# Connectome-based prediction of the severity of autism spectrum disorder

**DOI:** 10.1093/psyrad/kkad027

**Published:** 2023-11-27

**Authors:** Xuefeng Ma, Weiran Zhou, Hui Zheng, Shuer Ye, Bo Yang, Lingxiao Wang, Min Wang, Guang-Heng Dong

**Affiliations:** Department of Psychology, Yunnan Normal University, Kunming, Yunnan Province 650500, China; Center for Cognition and Brain Disorders, Hangzhou Normal University, Hangzhou, Zhejiang Province 311121, China; Zhejiang Key Laboratory for Research in Assessment of Cognitive Impairments, Hangzhou Normal University, Hangzhou, Zhejiang Province 311121, China; Institutes of Psychological Sciences, Hangzhou Normal University, Hangzhou, Zhejiang Province 311121, China; Center for Cognition and Brain Disorders, Hangzhou Normal University, Hangzhou, Zhejiang Province 311121, China; Institutes of Psychological Sciences, Hangzhou Normal University, Hangzhou, Zhejiang Province 311121, China; Shanghai Key Laboratory of Psychotic Disorders, Shanghai Mental Health Center, Shanghai Jiaotong University School of Medicine, Shanghai 200030, China; Kavli Institute for Systems Neuroscience, Centre for Neural Computation, Norwegian University of Science and Technology, Trondheim 7491, Norway; Center for Cognition and Brain Disorders, Hangzhou Normal University, Hangzhou, Zhejiang Province 311121, China; Institutes of Psychological Sciences, Hangzhou Normal University, Hangzhou, Zhejiang Province 311121, China; Center for Cognition and Brain Disorders, Hangzhou Normal University, Hangzhou, Zhejiang Province 311121, China; Zhejiang Key Laboratory for Research in Assessment of Cognitive Impairments, Hangzhou Normal University, Hangzhou, Zhejiang Province 311121, China; Department of Psychology, Yunnan Normal University, Kunming, Yunnan Province 650500, China; Department of Psychology, Yunnan Normal University, Kunming, Yunnan Province 650500, China

**Keywords:** autism spectrum disorder, connectome-based predictive modelling, resting-state functional connectivity, severity

## Abstract

**Background:**

Autism spectrum disorder (ASD) is characterized by social and behavioural deficits. Current diagnosis relies on behavioural criteria, but machine learning, particularly connectome-based predictive modelling (CPM), offers the potential to uncover neural biomarkers for ASD.

**Objective:**

This study aims to predict the severity of ASD traits using CPM and explores differences among ASD subtypes, seeking to enhance diagnosis and understanding of ASD.

**Methods:**

Resting-state functional magnetic resonance imaging data from 151 ASD patients were used in the model. CPM with leave-one-out cross-validation was conducted to identify intrinsic neural networks that predict Autism Diagnostic Observation Schedule (ADOS) scores. After the model was constructed, it was applied to independent samples to test its replicability (172 ASD patients) and specificity (36 healthy control participants). Furthermore, we examined the predictive model across different aspects of ASD and in subtypes of ASD to understand the potential mechanisms underlying the results.

**Results:**

The CPM successfully identified negative networks that significantly predicted ADOS total scores [*r* (df = 150) = 0.19, *P* = 0.008 in all patients; *r* (df = 104) = 0.20, *P* = 0.040 in classic autism] and communication scores [*r* (df = 150) = 0.22, *P* = 0.010 in all patients; *r* (df = 104) = 0.21, *P* = 0.020 in classic autism]. These results were reproducible across independent databases. The networks were characterized by enhanced inter- and intranetwork connectivity associated with the occipital network (OCC), and the sensorimotor network (SMN) also played important roles.

**Conclusions:**

A CPM based on whole-brain resting-state functional connectivity can predicted the severity of ASD. Large-scale networks, including the OCC and SMN, played important roles in the predictive model. These findings may provide new directions for the diagnosis and intervention of ASD, and maybe could be the targets in novel interventions.

## Introduction

Autism spectrum disorder (ASD) is a mosaic of developmental conditions characterized by early deficits in two behavioural domains: (i) difficulties in social communication and interaction and (ii) highly restricted, stereotypic, and repetitive behaviours (Yang, *et al*., 2023a, 2023b). The diagnosis of ASD is typically made through a comprehensive evaluation conducted by an experienced clinician. Currently, widely used observational measures for ASD diagnosis include the Autism Diagnostic Observation Schedule (ADOS) and the Autism Diagnostic Interview-Revised, which are considered the 'gold standard' in ASD-focused assessments. Additionally, a standard evaluation protocol often involves assessing IQ and adaptive behaviour (Braconnier & Siper, 2021).

Despite ongoing efforts, the diagnosis of ASD is still based on behavioural criteria. However, significant advances in identifying brain imaging biomarkers and developing tools to aid in the diagnosis of ASD have been made. Machine learning, a pattern recognition technique developed from artificial intelligence studies, has played a central role in this endeavour. The central tenet of machine learning is to automate inductive reasoning to create new knowledge (i.e. learning), a process achieved by extracting general rules and patterns from large datasets (Tai *et al*., 2019). Many studies have used similar machine-learning techniques with connectivity data in a classification framework to distinguish healthy control (HC) participants from patients, including those with ASD (Anderson *et al*., 2011; Plitt *et al*., 2015). Due to the high prevalence rate and heterogeneous nature of ASD, numerous machine-learning approaches have been developed over the last decade to investigate potential differences between ASD patients and neurotypical controls using functional magnetic resonance imaging (fMRI) data. However, a comprehensive understanding of the network basis underlying ASD and the intricate interactions between and within different brain networks is still lacking from the perspective of brain networks. Identifying brain-based predictive models for ASD will not only enhance our current biological understanding of ASD pathophysiology, which can further refine existing interventions, but may also have potential for direct application in real-world clinical practice.

Connectome-based predictive modelling (CPM) is a machine-learning, data-driven protocol that is used to develop predictive models of brain–behaviour relationships using whole-brain functional connectivity data (‘connectomes’) through cross-validation. Unlike traditional regression or correlation analyses, CPM does not necessitate *a priori* selection of networks, and it can identify specific regional connections associated with behaviours (Shen *et al*., 2017; Zhou *et al*., 2021). To ensure rigor and reproducibility, CPM incorporates a built-in cross-validation approach to avoid overfitting by testing model replication in a novel sample (Shen *et al*., 2017). This study used CPM to explore the brain network characteristics of participants with ASD for the following reasons: (i) CPM can reveal associations between brain connectivity patterns and behavioural outcomes or clinical diagnoses, which can be used to identify potential neural biomarkers and ASD-specific brain connectivity patterns. This facilitates ASD diagnosis, allowing it to move beyond reliance on behavioural criteria. (ii) CPM focuses on analysing the entire brain network rather than isolated brain regions. This allows researchers to understand the potential network basis and interactions underlying ASD from a large-scale brain network perspective. (iii) As a machine learning method, CPM may be used to discover previously unknown or unexpected brain–behaviour associations.

Some scholars have already applied CPM to research in the field of ASD. Lake *et al*.’s study on transdiagnostic social abilities related to ASD and attention-deficit/hyperactivity disorder (ADHD) applied CPM analysis to predict ADOS scores of ASD patients in the ABIDE-I/II datasets (Lake *et al*., 2019). Rohr *et al*. used CPM to predict scores on the Behavior Rating Inventory of Executive Function in participants from two sites in the Autism Brain Imaging Data Exchange II (ABIDE-II) and observed that the default mode network (DMN) played a significant role in alterations in inhibition and shifting (Rohr *et al*., 2020). Dufford *et al*. used CPM to predict the Social Responsiveness Scale scores of 144 participants in the Healthy Brain Network dataset, including 34 individuals with ASD (Dufford, 2022). They identified a common transdiagnostic social impairment network that was rooted in social areas of the brain, the subcortex, and the salience network.

Corresponding to the definition of ASD, the ADOS-G (generic) is a standardized, semistructured assessment that consists of four modules designed to observe social interaction, communication, play, language, and the imaginative use of materials by children, youth, and adults who may have ASD. The assessment includes 10–15 activities with 31 accompanying ratings, with questions related to social-emotional aspects and interview items about activities of daily living and additional tasks. Participants were tested at centres in the ABIDE using Modules 3 and 4 of the ADOS-G. Module 3 is intended for verbally fluent children in whom playing with toys is age-appropriate, while Module 4 is designed for verbally fluent adults and adolescents (usually over 12–16 years old) who do not have an interest in toys (Lord *et al*., 2000). The assessment provides a total score and subaspect scores, including social, communication, creativity, and stereotyped behaviour. This study aimed to explore the ability of CPM to predict ADOS total scores and subaspect scores in ASD patients.

In addition, ASD is part of a continuum of characteristics on a spectrum resulting from multiple nonlinear causative factors (Araujo, n.d.). Therefore, individuals with ASD exhibit a variety of clinical presentations, which makes the design of tests and subsequent interpretation of results challenging. According to the Diagnostic and Statistical Manual of Mental Disorders, fourth edition (DSM-IV), there are three subtypes of ASD: (i) classic autism (CA), (ii) Asperger's syndrome (AS), and (iii) pervasive developmental disorder not otherwise specified (PDD-NOS) (Lord, 2012). In the current study, we were also interested in whether the different subtypes of ASD show significant differences in CPM characteristics.

In the current study, we used dimensional CPM to identify neural networks that predicted the severity of ASD traits (measured by the ADOS) using whole-brain network data. Next, we conducted migration validation with an independent dataset to investigate the stability of the model and to identify any significant differences between ASD patients and HCs to test the specificity of the CPM prediction network. As many previous studies have suggested that social communication impairment is the most typical phenotype of ASD patients, we hypothesized that the brain networks associated with social cognition and communication would predict the ADOS scores of ASD patients.

## Methods

### Participants

The dataset in our study was obtained from the ABIDE-I/II. The scanning parameters at different sites in the ABIDE dataset vary; for details, please refer to the official website (http://fcon_1000.projects.nitrc.org/indi/abide/). To share resting-state functional MRI (rs-fMRI), anatomical, and phenotypic datasets with the broader scientific community, the ABIDE I involved 17 international sites and 1112 datasets, including 539 from individuals with ASD and 573 from HCs (aged 7–64 years, median age of 14.7 years across groups).

Our exclusion criteria for participants were as follows: (i) without functional or structural images; (ii) with mixed handedness or without handedness information; (ii) without full-scale intelligence quotient (FIQ) information or had an FIQ <70 (Floris *et al*., 2021); (iv) use of different parameters, such as eye status, repetition time, slice number, or data matrix size, from others used at that site; (v) use of time points different from others used at that site (time points varied between participants at the Stanford site, but all participants had at least 180 time points; consequently, 180 time points were ultimately used for all Stanford participants); (vi) signal losses revealed during visual inspection of functional images; (vii) scan duration <100 time points (Van Dijk *et al*., 2010); (viii) head motion exceeding 2 mm or 2°; (ix) bad spatial normalization revealed during visual inspection of functional images; (x) scan coverage of <91% of the whole brain; (xi) from sites with <20 individual datasets at each step (UCLA_2 after poor spatial normalization, Caltech after inadequate cover); (xii) a DSM-IV score of −9999 (an invalid score); (xiii) without ADOS scores; or (xiv) mean framewise displacement (mFD) is >0.2 mm. Ultimately, a total of 359 participants, including 323 ASD patients (151 in model sample and 172 in validation sample) and 36 HCs, were included in our investigation. The demographic information of the included participants is shown in Table 1.

**Table 1:** Demographic: information.

**Model sample (*n* = 151)**	
**Gender (male/female)**	142/9
**Age (mean ± SD, years)**	17.02 ± 6.68
**mFD (mean ± SD, mm)**	0.08 ± 0.04
**ADOS scores**	
** Total**	11.89 ± 4.00
** Social interaction**	8.12 ± 2.94
** Communication**	3.93 ± 1.62
** Stereotyped behaviours**	2.10 ± 1.78
**Validation sample (*n* = 172)**	
**Gender (male/female)**	156/16
**Age (mean ± SD, years)**	17.02 ± 10.01
**mFD (mean ± SD, mm)**	0.10 ± 0.05
**ADOS scores**	
** Total**	10.38 ± 3.91
** Social interaction**	7.05 ± 2.59
** Communication**	3.08 ± 1.50
** Stereotyped behaviours**	1.71 ± 1.38
**HCs (*n* = 36)**	
**Gender (male/female)**	35/1
**Age (mean ± SD, years)**	20.92 ± 5.35
**mFD (mean ± SD, mm)**	0.10 ± 0.04
**ADOS scores**	
** Total**	1.33 ± 1.66
** Social interaction**	0.67 ± 0.96
** Communication**	0.67 ± 1.01
** Stereotyped behaviours**	0.03 ± 1.67

### MRI data preprocessing

**Figure 1:**
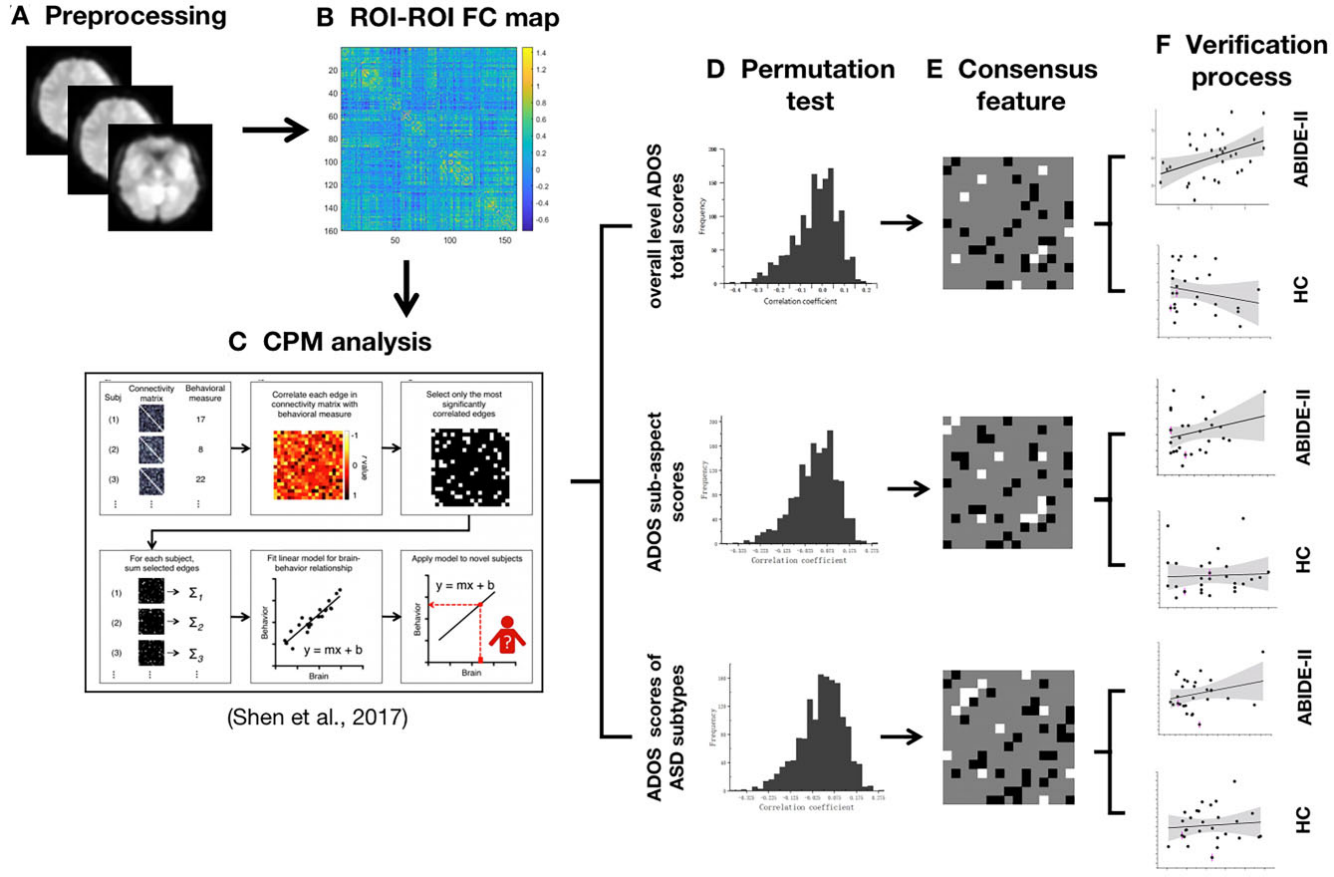
Diagrams of the analysis process of this study. (A) The fMRI data were obtained from ABIDE-I and ABIDE-II, and all the data were preprocessed using SPM 12 and DPABI. (B) The subplot shows the calculation of task-related ROI–ROI functional connectivity based on the Dosenbach 160 ROI functional template. (C) Schematic of CPM. (D) Permutation testing was used 1000 times to examine the performance of the models. (E) The consensus features of models with significant predictive effects were extracted. (F) To evaluate the replicability and specificity of the models, the networks were migrated to new independent datasets for the verification process.

Figure 1 depicts the research process. MRI data were preprocessed with Statistical Parametric Mapping (SPM) v.12 (http://www.fil.ion.ucl.ac.uk/spm/software/spm12/) and DPABI V5.1 (http://rfmri.org/dpabi). The preprocessing steps included the following: (i) discarding the first 10 time points, (ii) slice timing and head motion correction, (iii) spatial normalization with the forwards transformation field into the standard Montreal Neurological Institute (MNI) space (resampling voxel size was 3 × 3 × 3 mm^3^), (iv) spatial smoothing with a 6 mm full-width at half-maximum Gaussian kernel, (v) removal of the linear trends of time courses, (vi) adjustment for nuisance covariates (i.e. head motion covariates with the Friston 24-parameter model as well as white matter and cerebrospinal fluid signals), (vii) each time point where the framewise displacement (FD) exceeded 0.2 mm was used as a separate regressor for scrubbing, and (viii) application of a bandpass temporal filter (0.01–0.08 Hz) to the time series (Fig. 1A).

### Functional network construction

Whole-brain resting-state functional connectivity (rsFC) was calculated using the GRETNA Toolbox (https://www.nitrc.org/projects/gretna/) with the Dosenbach atlas, which consists of 160 regions of interest (ROI). Every ROI in the atlas was a spherical region with a 5 mm radius, and all regions were assigned into six predefined networks: the DMN, frontoparietal network, cingulate-opercular network, sensorimotor network (SMN), occipital network (OCC), and cerebellum network (Dosenbach *et al*., 2010). By averaging the time courses of all voxels within a node, we extracted the time courses of all nodes and calculated Pearson's correlation coefficients between them. The results were then transformed using Fisher's *z*-transformation, resulting in a 160 × 160 correlation matrix for each participant in which each edge represented the rsFC strength between two nodes (Fig. 1B).

### Multisite effect correction

By using the ComBat function available in MATLAB (https://github.com/Jfortin1/ComBatHarmonization), we removed the site effects to account for site, collection time, and data acquisition parameter variability across each of the collection centres in the ABIDE I. A previous study showed that this approach can effectively account for scanner-related variance in multisite rfMRI datasets. The default ComBat function uses a nonparametric empirical Bayes procedure with a prior and treats the diagnosis, age, sex, FIQ, and mFD as biological variables of interest.

### Connectome-based predictive modelling

As Fig. 1C shows, all analyses used CPM (https://www.nitrc.org/projects/bioimagesuite) to estimate predictive models based on whole-brain rsFC. We applied the Dosenbach 160 ROI functional template and calculated the ROI–ROI functional connectivity. First, after controlling for confounding variables (i.e. age, sex, and head motion), correlation coefficients between each edge and a vector of behavioural values (here, ADOS scores) were calculated. A threshold (*P* < 0.01) was applied to the matrix to retain edges that were important for the subsequent processing. The positive and negative networks consisted of edges that were significantly positively (negatively) correlated with ADOS scores. Next, the sum of edge weights (connectivity strength) in positive and negative networks was calculated for each participant to form single-subject positive and negative network strengths. The positive and negative network strengths were then included as regression factors to construct linear regression predictive models for predicting ADOS scores. Finally, linear regression predictive models were used on data from new participants to generate predicted values.

### Cross-validation

To ensure independence between feature selection and prediction, leave-one-out cross-validation was applied to the model. In this approach, each participant's predicted value (i.e. the ‘left-out’ subject) was set as the testing set, while all other participants' predicted values were set as the training set so that all participants had a predicted value according to this iterative manner. Edges that meet the predefined threshold (in this study, *P* < 0.01) are treated as key edges during each iteration. Those edges that are consistently retained across all iterations will be marked in the final network (consensus features), which is a matrix composed of 0, 1, and −1. Here, 1 represents key edges in the positive network, and −1 represents key edges in the negative network. In addition, we also computed the partial correlation coefficient (*r*) between the model's predicted values and the actual values, taking sex, age, and mFD as covariates. The significance of *r* was assessed based on null distributions, which were generated by randomly shuffling the correspondence between behavioural variables and connectivity matrices 1000 times and repeating the CPM analysis with the shuffled data. We used the average of the resulting 1000 correlation coefficients to represent the average model performance (Fig. 1D).

### Replication of the predictive networks across datasets

CPM analyses were used to calculate connectomes from new ASD datasets to predict ADOS scores. Edge weights corresponding to ASD networks were then extracted from connectomes (Fig. 1E). To assess the replicability and specificity of the networks across different datasets, we validated the outcome networks into a independent clinical sample (172 ASD patients from ABIDE-II dataset) and a healthy sample (36 HCs with ADOS scores). We used partial correlation analysis to examine the relationship between predicted values and actual values (Fig. 1F).

## Results

### CPM for predicting ADOS total scores at the overall level

**Figure 2:**
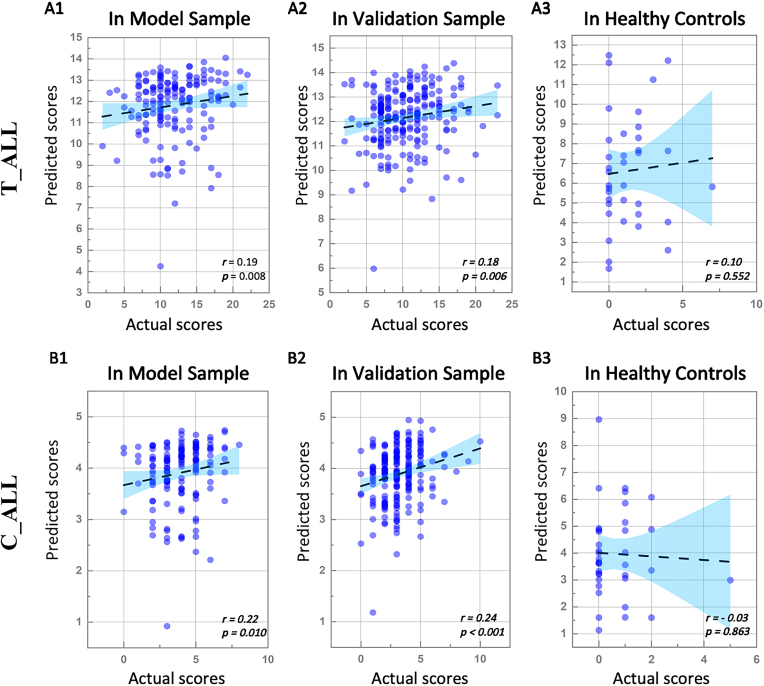
Model performance among all patients. Abbreviations: T_CA, the negative network model for predicting ADOS total scores of all patients; C_CA, the negative network model for predicting ADOS communication scores of all patients. (A1) and (B1) are the partial correlation scatterplots of the predicted values and the actual values in the model sample, demonstrating that both T_CA and C_CA exhibit great predictive power. (A2) and (B2) are the partial correlation scatterplots of the predicted values and the actual values in the validation sample, demonstrating that both T_CA and C_CA are replicable in independent clinical samples. (A3) and (B3) are the partial correlation scatterplots of the predicted values and the actual values in HCs, proving the relative specificity of T_CA and C_CA.

Among all participants, the CPM results demonstrated that negative network significantly predicted ADOS total scores [*r* (df = 150) = 0.19, *P* = 0.008] (Fig. 2A1), whereas positive network did not [*r* (df = 150) = −0.004, *P* = 0.540]. After establishing the models, we applied their consensus feature to perform another CPM analysis of independent clinical data and HCs for verification. The negative network successfully predicted the ADOS total score in the validation sample [*r* (df = 171) = 0.18; *P*_FDR_ = 0.006] (Fig. 2A2). Thus, the ability of the identified networks to predict ADOS total scores in a separate, heterogeneous replication sample was acceptable. To verify the specificity of the networks, we applied the models to predict ADOS scores in HCs. Partial correlation analyses indicated no significant associations between predicted values and actual values of HCs [*r* (df = 35) = 0.10, *P*_FDR_ = 0.552] (Fig. 2A3). The results suggest that the identified networks significantly predicted ADOS total scores among participants with ASD.

### CPM for predicting ADOS subaspect scores

The CPM results demonstrated that negative network significantly predicted ADOS communication scores [*r* (df = 132) = 0.22, *P* = 0.010] (Fig. 2B1) but not ADOS social interaction scores [*r* (df = 132) = 0.12, *P* = 0.096] or ADOS stereotyped behaviour scores [*r* (df = 132) = 0.12, *P* = 0.140]. Positive network significantly predicted ADOS social interaction scores [*r* (df = 132) = 0.21, *P* = 0.009] but not ADOS communication scores [*r* (df = 132) = −0.18, *P* = 0.710] or ADOS stereotyped behaviour scores [*r* (df = 132) = −0.02, *P* = 0.400]. Due to the lack of data on creativity scores in the ABIDE dataset, we did not perform the corresponding calculations in this study. The negative network for predicting ADOS communication scores reached statistical significance in the validation [*r* (df = 171) = 0.24, *P* < 0.001] (Fig. 2B2). The positive network predicting social interaction scores was not replicated [*r* (df = 171) = −0.09, *P* = 0.561]. In addition, partial correlation analyses indicated no significant associations between predicted values and actual values of the negative network for predicting ADOS communication scores in HCs [*r* (df = 35) = −0.03, *P* = 0.863] (Fig. 2B3).

### CPM for predicting ADOS scores in different ASD subtypes

**Figure 3:**
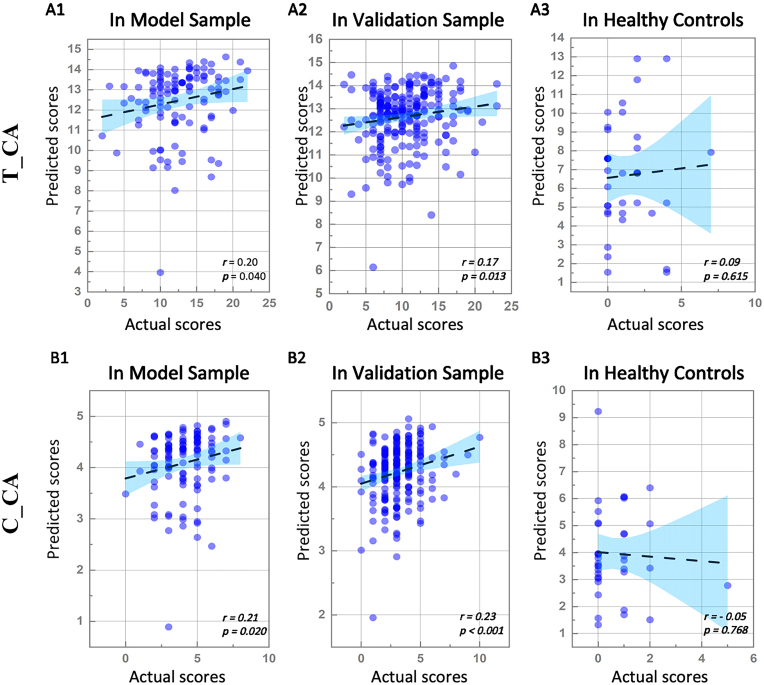
Model performance among patients with CA. Abbreviations: T_CA, the negative network model for predicting ADOS total scores of patients with CA; C_CA, the negative network model for predicting ADOS communication scores of patients with CA. (A1) and (B1) are the partial correlation scatterplots of the predicted values and the actual values in the model sample, demonstrating that both T_CA and C_CA exhibit great predictive power. (A2) and (B2) are the partial correlation scatterplots of the predicted values and the actual values in the validation sample, demonstrating that both T_CA and C_CA are replicable in independent clinical samples. (A3) and (B3) are the partial correlation scatterplots of the predicted values and the actual values in HCs, proving the relative specificity of T_CA and C_CA.

Among participants with CA, the CPM results demonstrated that negative network significantly predicted ADOS total scores [*r* (df = 104) = 0.20, *P* = 0.040] (Fig. 3A1), ADOS social interaction scores (*r* [df = 104] = 0.16, *P* = 0.030) and ADOS communication scores [*r* (df = 104) = 0.21, *P* = 0.020] (Fig. 3B1) but not ADOS stereotyped behaviour scores. By contrast, positive network did not reliably predict any of these scores. In the 30 AS participants, CPM did not yield any effective predictive networks. Notably, we did not calculate the CPM among participants with PDD-NOS because the sample size was too small. The negative networks for predicting ADOS total [*r* (df = 171) = 0.17, *P*_FDR_ = 0.013] (Fig. 3A2) and communication [*r* (df = 171) = 0.23, *P*_FDR_ < 0.001] (Fig. 3B2) scores among CA patients reached statistical significance in the validation sample. The negative network predicting social interaction scores for CA patients was not replicated [*r* (df = 171) = 0.11, *P* = 0.141]. Partial correlation analyses indicated no significant associations between predicted values and actual values of the negative network for predicting ADOS total (*r* (df = 35) = 0.09, *P* = 0.615] (Fig. 2A3) and communication [*r* (df = 35) = −0.05, *P* = 0.768] (Fig. 3B3) scores in HCs.

### Network anatomy and overlap with canonical neural networks

Finally, four negative networks for predicting ADOS total and communication scores, both for the entire sample and specifically for CA patients, were found to be effective after validation in independent samples and HCs. For the convenience of discussions, we will refer to these four network models as T_CA, C_CA, T_CA, and C_CA respectively. To further mitigate the potential influence of head motion, we excluded participants with mFD >0.15 mm and obtained similar results. These findings are included in the supplementary materials.

**Figure 4:**
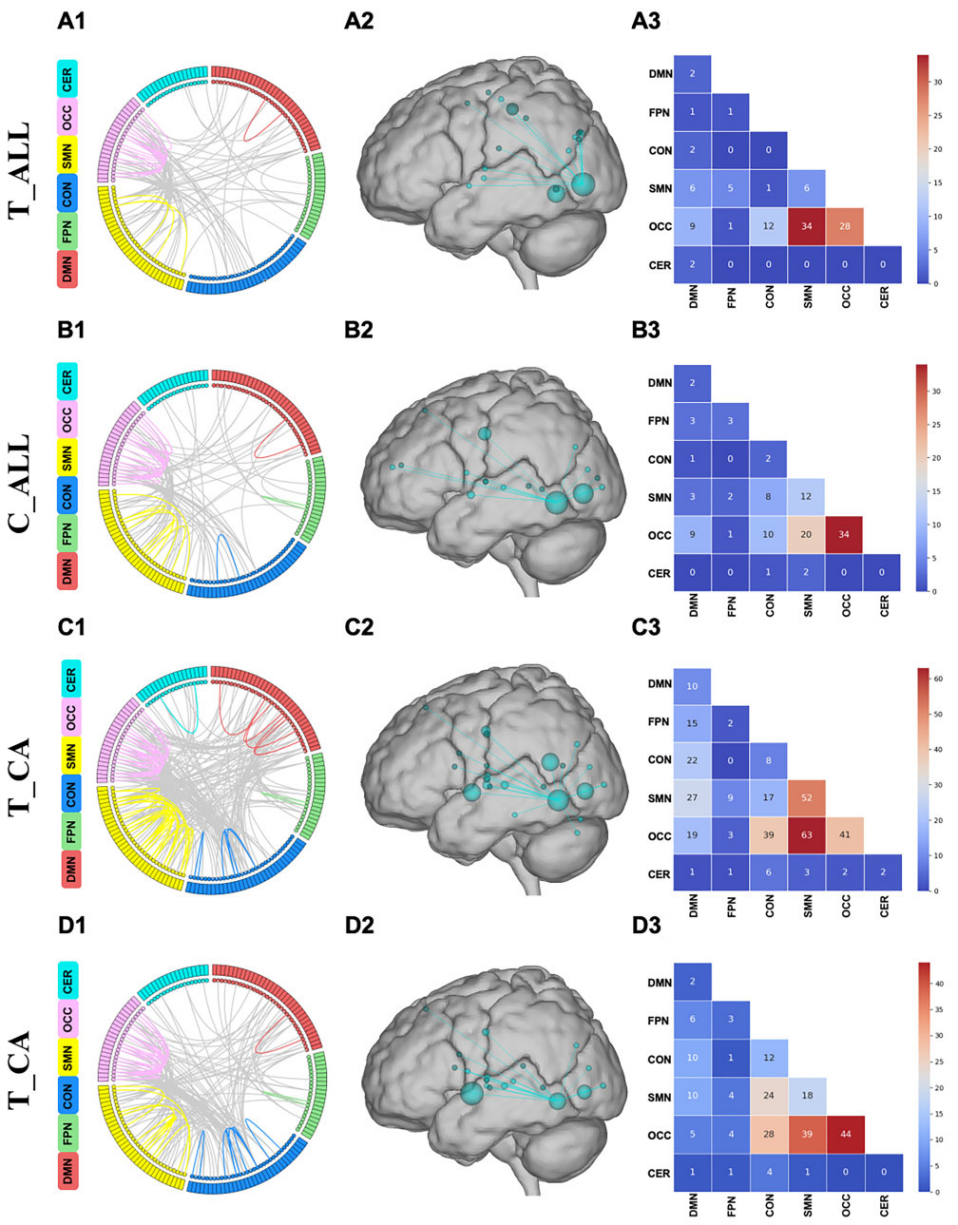
Key nodes and networks of each model. (A), (B), (C), and (D), respectively, illustrate the network anatomy and overlap of T_ALL, C_ALL, T_CA, and C_CA. (1) The circle plot shows the negative network connections among 160 nodes, which were divided into six brain networks according to the Dosenbach 160 ROI functional template and colour coded. (2) The glass brain plot shows the key nodes (circles) and edges (lines, representing functional connections) in the negative network, and the size of the nodes reflects the number of connections related to the node. (3) The matrix plot shows intra- and internetwork connectivity in the negative network.

Across all folds of cross-validation, 110 negative edges were retained as consensus functional connections of T_CA and included connections within and between multiple macroscale brain regions (e.g. frontal, parietal, occipital, temporal, limbic, and cerebellar regions) (Fig. 4A1). The nodes with the most connections in the negative network included a postoccipital node (Node no. 134, 17°), a temporal node (Node no. 123, 13°), an anterior cingulate cortex node (Node no. 137, 10°), and an inferior parietal lobe node (Node no. 117, 9°) (Fig. 4A2). According to the results, the connections between the OCC and SMN and intra-network connections of OCC contributed most to T_CA (Fig. 4A3). Regarding the C_CA, 123 negative edges were retained as consensus functional connections (Fig. 4B1). The nodes with the most connections in the negative network are similar to T_CA, including the temporal node (Node no. 123, 17°), the postoccipital node (Node no. 134, 13°), and a precentral gyrus node (Node no. 101, 10°) (Fig. 4B2). At the network level, the connections between the OCC and SMN and intra-network connections of OCC contributed most to C_CA (Fig. 4B3).

For participants with CA, the CPM results reveal a more extensive overlap of neural networks; 342 negative edges were retained as consensus functional connections of T_CA (Fig. 4C1). The nodes with the most connections in the negative network included a post-parietal node (Node no. 117, 26°), an occipital node (Node no. 123, 24°), a mid-insula node (Node no. 69, 20°), and a post-cingulate node (Node no. 24, 20°) (Fig. 4C2). On the basis of the results, OCC and SMN exhibit the most intra-network connections, whereas OCC with SMN and OCC with CON exhibit the most inter-network connections. The DMN has more connections with networks other than the cerebellum network, especially with the previously mentioned OCC, SMN, and CON (Fig. 4C3). Next, 217 negative edges were retained as consensus functional connections of C_CA (Fig. 4D1). The nodes with the most connections in the negative network included a mid-insula node (Node no. 69, 27° and two occipital nodes (Node nos 122 and 123, 20°) (Fig. 4D2). At the network level, some intra-network connections (OCC-SMN, OCC-CON, and SMN-CNN) and intra-network connections of OCC contributed most to C_CA (Fig. 4D3).

## Discussion

In this study, we used a data-driven, connectome-based machine learning protocol to predict ADOS scores. Our research findings indicate that CPM successfully predicted the ADOS total and communication scores for ASD patients, both in all patients and in the CA subtype. Moreover, the validation across independent clinical datasets and in HCs provides strong evidence for the replicability of our identified networks. These results will deepen our understanding of how functional connectivity coalesces to give rise to the complex autism syndrome and may assist in clinical interventions and treatments (Horien *et al*., 2022).

### Critical networks in predicting ADOS total scores

Our study highlighted the importance of connections related to the OCC in predicting ADOS total scores in ASD patients. Numerous studies have demonstrated that patients with ASD exhibit atypical sensory processing, including both hypersensitivity and hyposensitivity (Baron-Cohen *et al*., 2009; Bast, 2007; Baum *et al*., 2015). In several sensory domains, individuals often complain of visual symptoms (Simmons *et al*., 2009), and these symptoms increase the burden of depression (Bitsika *et al*., 2016). A previous study revealed that patients with ASD exhibit abnormal activation in the dorsolateral prefrontal cortex, insula, posterior medial prefrontal cortex, and occipital cortex compared to typically developing individuals (Di Martino *et al*., 2014), suggesting the presence of visual processing deficits in ASD patients. Therefore, our results suggest that both the intranetwork connectivity of the OCC and specific interactions between the OCC and other networks may impair the integration of visual information, which in turn relates to atypical social communication in individuals with ASD. However, this speculative conclusion requires verification in a longitudinal sample to establish its validity.

Additionally, the SMN played a crucial role in predicting ADOS total scores in our model. Sensorimotor disturbances are considered a core symptom of ASD in the DSM-5 and persist throughout the lifespan. These disturbances include sensory disturbances, clumsiness, postural instability, and impaired visuomotor coordination (Fournier *et al*., 2010). Moreover, motor impairments in gross and fine motor skills, as well as in socially embedded motor skills such as imitation and praxis, have been observed in individuals with ASD (Amonkar *et al*., 2021). These motor dysfunctions have been associated with the clinical symptomology (Sutera *et al*., 2007) and sociobehavioural traits (MacDonald *et al*., 2013) of ASD patients. Several researchers have examined the association between motor impairment and the severity of ASD core symptoms in the areas of social communication, repetitive behaviours, and cognition (Amonkar *et al*., 2021; MacDonald *et al*., 2013). Brian *et al*. used factor analysis and revealed significant correlations between restricted behaviours and hyperresponsive sensory symptoms in children with ASD (Boyd *et al*., 2010). Penelope *et al*. also revealed significant correlations between sensory profiles and Autism Diagnostic Interview-Revised restricted and repetitive behaviour (RRB) scores (Hannant *et al*., 2016). We speculate that the patterns of connectivity between the SMN and other networks in the ASD network may impair sensorimotor information integration and are related to atypical social communication in ASD patients. Therefore, to some extent, the increased connectivity of the SMN may be a neural signature of ASD that can be used for prediction and diagnosis.

Our study revealed numerous connections both between networks and within networks, prominently involving the OCC and SMN, which is consistent with previous research (Rohr *et al*., 2020). These findings raise questions about the involvement of the parietal and occipital lobes in the psychopathology of autism. Rohr *et al*. found that functional connectivity abnormalities in the SMN and visual networks were related to impaired inhibitory control in children with ASD (Rohr *et al*., 2020). Heng Chen *et al*. also found increased connectivity of the SMN and visual networks in ASD, and the insular cortex and occipital cortex were the most connected regions (Chen *et al*., 2018). Applying functional connectivity analyses of intra- and internetwork connectivity, Bosi Chen *et al*. reported increased network connectivity between regions in the visual network and SMN in the ASD group. This visual-sensory-motor hyperconnectivity is particularly noteworthy in light of the sensory processing abnormalities and multisensory integration impairments in ASD. Additionally, this hyperconnectivity was correlated with heightened ASD symptomatology (Chen *et al*., 2021). These findings may indicate inadequate integration of visual and somatosensory input into the socioaffective circuits, which in turn affects social interactions.

Overall, the OCC, responsible for visual processing, plays a fundamental role in recognizing and interpreting facial expressions, body language, and other nonverbal cues, which are essential for social interaction and understanding emotions (Peelen *et al*., 2010). Similarly, the SMN, which is involved in motor planning and execution, is also closely linked to social cognition, as it underlies the ability to imitate and mirror others' actions, fostering social learning and empathy (Hamilton, 2013). The heightened connectivity in these networks might indicate an altered neural mechanism for social information processing in ASD, contributing to the difficulties observed in social communication and interaction. Further studies are needed to examine the relationships between activity in these networks and symptom severity to support the hypothesis that visual-sensory-motor hyperconnectivity may underlie the atypical social interaction and verbal communication observed in ASD.

### The predictive role of communication features in ASD diagnosis

When predicting the communication scores for all ASD patients, we obtained a network structure that closely resembled the negative network model for predicting total scores, and this was also the case for CA patients. Furthermore, while social interaction scores were also predictible, they could not be validated in an independent clinical sample, and this model retained only a few key edges. Impaired communication is one of the core features of ASD patients. ASDs coexist with other communication difficulties such as a language disorder, apraxia of speech, speech sound disorders, and/or other neurodevelopmental disorders. Features and clinical markers associated with these coexisting conditions can be used for differential diagnosis and various interventions (Vogindroukas *et al*., 2022). Our findings may provide neurological evidence for that impaired communication might be a key feature in predicting the severity of ASD.

### Differences in predicting ASD subtypes

In terms of subtypes, CA patients were able to successfully predict both total and communication scores and validate them in an independent sample, whereas AS patients were unable to derive a predictive model. Due to the insufficient number of PDD-NOS patients in the sample, this study did not include a discussion on it. It should also be noted that CA is perhaps the broadest and most predominant form of ASD subtypes, and in our study, CA patients constituted the majority of the entire sample (115/157, 73%).

Differing from T_CA and C_CA, in T_CA and C_CA, the role of network connections involved in DMN and CON are emphasized. In ASD, altered functional connectivity involving regions in the DMN has been confirmed to be associated with inhibition (Voorhies *et al*., 2018) and Social Responsiveness Scale scores (Jann *et al*., 2015). The DMN is known to exhibit high activity during the resting state and suppressed activity during cognitively demanding tasks. Furthermore, since the DMN is also associated with a state of readiness for environmental changes, it may be responsible for the poor awareness of and response to social environments in ASD patients. The CON (also referred to as the salience or ventral attention network) is commonly referred to as 'cognitive control' networks, and is crucial for an individual's executive functions (Hausman *et al*., 2021; Sadaghiani & D'esposito, 2015). Disruptions in the CON have been linked to several neuropsychiatric conditions. Raichle and Snyder discussed the involvement of this network in disorders such as ADHD, emphasizing its role in the regulation of attention (Raichle & Snyder, 2007). Kennedy and Courchesne *et al*. suggest that alterations in the CON might have potential contributions to the communication and social difficulties observed in individuals with ASD (Kennedy & Courchesne, 2008). Furthermore, T_CA and C_CA exhibit a broader overlap of neural networks. Considering that all models are ultimately negative networks, meaning that the lower severity of the participants, the less close the functional connections involved in these networks. Therefore, we believe that the reduction in brain functional network connectivity may lead to the attention and executive function deficits and motor coordination issues, as well as social and emotional regulation deficits commonly observed in CA patients.

### Limitations

Several limitations should be noted. First, after our screening process, only a limited number of participants remained for model training and testing, and the total number of participants with PDD-NOS was insufficient in this subset. Additionally, almost all participants lacked ADOS creativity scores, which led to the omission of corresponding calculations in this study. The examination of network characteristics in different subtypes and subaspect scores was incomplete, and more participants are needed to test the generalizability of the predictive models. Second, the DSM-5 explicitly recognizes that ASD can coexist with other disorders, including genetic disorders (e.g. fragile X syndrome) and psychiatric conditions (e.g. ADHD). The CPM network obtained in this study should be explored in subsequent studies to clarify its specificity in ASD patients with comorbities. Last but not the least, nonlinear effects of movement could persist even after extensive motion correction (Shen *et al*., 2017). Further research is needed to explore the impact of head motion on CPM results.

## Conclusions

The current study showed that a connectome comprising whole-brain rsFC effectively predicted ADOS total and communication scores among all ASD patients or CA subtype. Notably, the high-degree nodes in the predictive model were primarily located in the occipital cortex and sensorimotor cortex, and the model involved connections between and within multiple well-established neural networks. The CPM approach provides a novel and valuable method of understanding the neural underpinnings of ASD traits, and the findings may have potential applications for the diagnosis and intervention of ASD.

## Supplementary Material

kkad027_Supplemental_File

## Data Availability

The data that support the findings of this study are available from the corresponding author upon reasonable request. The script and some of the data used in this study have been uploaded to https://github.com/Alainfuren/CPM_ASD.
