# Supplementary material for: Connectome-based prediction of the severity of autism spectrum disorder
Source: Psychoradiology. 2023 Nov 27;3:kkad027. doi: 10.1093/psyrad/kkad027 (PMC10917386; doi:10.1093/psyrad/kkad027)
Supplement: kkad027_Supplemental_File [file kkad027_Supplemental_File.docx]

**Supplementary materials for:**

**Connectome-based prediction of the severity of autism spectrum disorder**

**Demographic information for subjects with mFD <0.15mm**

To further mitigate the potential influence of head motion, we excluded participants with mFD greater than 0.15 mm. The demographic information of the included subjects is shown in Table S1.

| **Model Sample (n=139)** | | |
| --- | --- | --- |
| **Gender (male/female)** |  | 130/9 |
| **Age (mean ± SD,years)** |  | 16.99±6.72 |
| **mFD (mean ± SD,mm)** |  | 0.08±0.03 |
| **ADOS scores** |  |  |
| **Total** |  | 11.72±4.00 |
| **Social Interaction** |  | 7.96±2.93 |
| **Communication** |  | 3.91±1.65 |
| **Stereotyped Behaviors** |  | 2.03±1.79 |
| **Validation Sample (n=143)** | | |
| **Gender (male/female)** |  | 129/14 |
| **Age (mean ± SD,years)** |  | 16.65±10.34 |
| **mFD (mean ± SD,mm)** |  | 0.09±0.03 |
| **ADOS scores** |  |  |
| **Total** |  | 10.20±3.94 |
| **Social Interaction** |  | 7.00±2.64 |
| **Communication** |  | 3.00±1.48 |
| **Stereotyped Behaviors** |  | 1.70±1.39 |

**Table S1**. Abbreviations: ABIDE, the Autism Brain Imaging Data Exchange; mFD, mean framewise head displacement; HC, Healthy Control; ADOS, the Autism Diagnostic Observation Schedule.

**CPM results using subjects with mFD <0.15**

Among all subjects, the CPM results demonstrated that negative network significantly predicted ADOS total scores (*r* [df = 138] = 0.17, *p* = 0.020), whereas positive network did not. The negative network successfully predicted the ADOS total score in the validation sample (*r* [df = 142] = 0.15; *p*_FDR_ = 0.047). Partial correlation analyses indicated no significant associations between predicted values and actual values of HCs (*r* [df = 35] = -0.14, *p*_FDR_ = 0.612). Only one negative network significantly predicted ADOS communication scores (*r* [df = 122] = 0.19, *p* = 0.010) but not ADOS social interaction scores (*r* [df = 122] = 0.10, *p* = 0.127) or ADOS stereotyped behaviour scores (*r* [df = 118] = 0.02, *p* = 0.670). In contrast, positive network did not reliably predict any of these scores. The negative network for predicting ADOS communication scores reached statistical significance in the validation (*r* [df = 142] = 0.25, *p* < 0.001). In addition, partial correlation analyses indicated no significant associations between predicted values and actual values of the negative network for predicting ADOS communication scores in HCs (*r* [df = 35] = 0.07, *p* = 0.402).

Among subjects with CA, the CPM results demonstrated that negative network significantly predicted ADOS total scores (*r* [df = 94] = 0.20, *p* = 0.010), ADOS social interaction scores (*r* [df = 94] = 0.15, *p* = 0.044) and ADOS communication scores (*r* [df = 94] = 0.19, *p* = 0.040). In contrast, positive network did not reliably predict any of these scores. In the 27 AS participants, CPM did not yield any effective predictive networks. The negative networks for predicting ADOS total (*r* [df = 142] = 0.20, *p*_FDR_ = 0.002) and communication (*r* [df = 142] = 0.24, *p*_FDR_ < 0.001) scores among CA patients reached statistical significance in the validation sample. The negative network predicting social interaction scores demonstrates a significant trend in the validation sample (*r* [df = 142] = 0.15, *p* = 0.047). Partial correlation analyses indicated no significant associations between predicted values and actual values of the negative network for predicting ADOS total (*r* [df = 35] = 0.09, *p* = 0.615) and communication (*r* [df = 35] = -0.05, *p* = 0.768) scores in HCs. All the results are visually presented in Table S2.

|  | **TOTAL** | **SOCIAL** | **COMM** | **STERO** |
| --- | --- | --- | --- | --- |
| **ALL** | Pos: ns Neg: r=0.17, p=0.020 Val: r=0.15, p=0.047 HCs: ns | Pos: ns Neg: ns | Pos: ns Neg: r=0.19, p=0.010 Val: r=0.25, p<0.001 HCs: ns | Pos: ns Neg: ns |
| **CA** | Pos: ns Neg: r=0.20, p=0.010 Val: r=0.20, p=0.003 HCs: ns | Pos: ns Neg: r=0.17, p=0.020 Val: r=0.15,p=0.047 HCs: ns | Pos: ns Neg: r=0.19, p=0.040 Val: r=0.24, p<0.001 HCs: ns | Pos: ns Neg: ns |
| **AS** | Pos: ns Neg: ns | Pos: ns Neg: ns | Pos: ns Neg: ns | Pos: ns Neg: ns |

**Table S2**. Abbreviations: TOTAL, the Autism Diagnostic Observation Schedule (ADOS) total socres; SOCIAL, the ADOS social interaction scores; COMM, the ADOS communication scores; STERO, the ADOS stereotyped behaviour scores; ALL, all patients of the model sample; CA, the classic autism patients of the model sample; AS, the Asperger’s syndrome patients of the model sample; Pos, positive network performence; Neg, negative network performence; Val, network performence in validation sample; HCs, network performence in healthy controls; ns, not significant.

In line with the results in the main manuscript, T_CA, C_CA, T_CA, and C_CA were retained in the final analysis. Across all iterations of cross-validation, we retained 67 negative edges as consensus functional connections for T_ALL, 76 negative edges for C_ALL, 136 negative edges for T_CA, and 130 negative edges for C_CA. The network anatomy and overlap with canonical neural networks are shown in Figure S1.

**
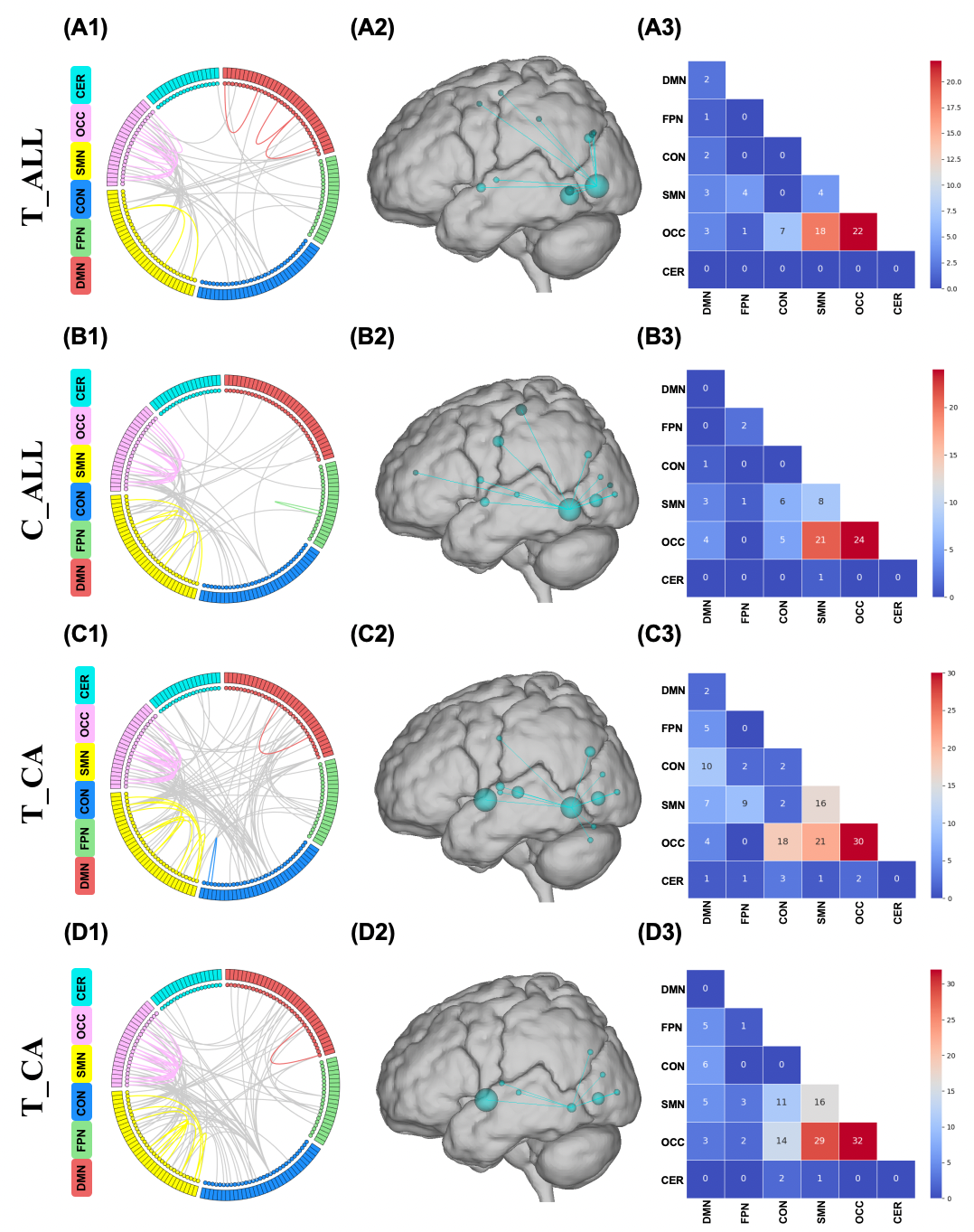
**

**Figure S1**. **Key nodes and networks of each model.** (A), (B), (C), and (D) respectively illustrate the network anatomy and overlap of T_ALL, C_ALL, T_CA, and C_CA. (1) The circle plot shows the negative network connections among 160 nodes, which were divided into 6 brain networks according to the Dosenbach 160 ROI functional template and colour coded. (2) The glass brain plot shows the key nodes (circles) and edges (lines, representing functional connections) in the negative network, and the size of the nodes reflects the number of connections related to the node. (3) The matrix plot shows intra- and internetwork connectivity in the negative network.

While the predictive performance of the four models differed slightly at thresholds of 0.2 and 0.15, overall, the CPM results did not exhibit significant changes due to head motion control, especially in maintaining stability in the overlap between key nodes and brain networks. In conclusion, these results proved that connectome comprising whole-brain rsFC could effectively predicted ADOS total and communication scores among all ASD patients or CA subtype. The connections between the OCC and SMN and intra-network connections of OCC contributed most to the models.

However, nonlinear effects of movement could persist even after extensive motion control. We observed that with the decrease in the mFD threshold, there is a trend of reduced consensus features retained by the models. Due to the variation in sample size caused by the mFD threshold, the predicted networks produced by CPM are essentially different. Therefore, further research is warranted to explore the impact of head motion control on CPM results and the practical implications behind this influence.
